# Recognizing and Counting Freehand Exercises Using Ubiquitous Cellular Signals †

**DOI:** 10.3390/s21134581

**Published:** 2021-07-04

**Authors:** Guanlong Teng, Yue Xu, Feng Hong, Jianbo Qi, Ruobing Jiang, Chao Liu, Zhongwen Guo

**Affiliations:** Department of Computer Science and Technology, Ocean University of China, Qingdao 266100, China; tgl@stu.ouc.edu.cn (G.T.); xuyue@stu.ouc.edu.cn (Y.X.); qijianbo2018@stu.ouc.edu.cn (J.Q.); jrb@ouc.edu.cn (R.J.); liuchao@ouc.edu.cn (C.L.); guozhw@ouc.edu.cn (Z.G.)

**Keywords:** cellular signal, freehand exercise, wireless sensing, mobile sensing, cellular sensing

## Abstract

Freehand exercises help improve physical fitness without any requirements for devices or places. Existing fitness assistant systems are typically restricted to wearable devices or exercising at specific positions, compromising the ubiquitous availability of freehand exercises. In this paper, we develop MobiFit, a contactless freehand exercise assistant using just one cellular signal receiver placed on the ground. MobiFit passively monitors the ubiquitous cellular signals sent by the base station, which frees users from the space constraints and deployment overheads and provides accurate repetition counting, exercise type recognition and workout quality assessment without any attachments to the human body. The design of MobiFit faces new challenges of the uncertainties not only on cellular signal payloads but also on signal propagations because the sender (base station) is beyond the control of MobiFit and located far away. To tackle these challenges, we conducted experimental studies to observe the received cellular signal sequence during freehand exercises. Based on the observations, we constructed the analytic model of the received signals. Guided by the insights derived from the analytic model, MobiFit segments out every repetition and rest interval from one exercise session through spectrogram analysis and extracts low-frequency features from each repetition for type recognition. Extensive experiments were conducted in both indoor and outdoor environments, which collected 22,960 exercise repetitions performed by ten volunteers over six months. The results confirm that MobiFit achieves high counting accuracy of 98.6%, high recognition accuracy of 94.1% and low repetition duration estimation error within 0.3 s. Besides, the experiments show that MobiFit works both indoors and outdoors and supports multiple users exercising together.

## 1. Introduction

Freehand exercise, with its advantage of convenience, has become one of the most popular physical activities to improve fitness, reduce weight and keep in shape [1]. The assistant to track and assess freehand exercises helps improve fitness quality. However, existing fitness assistants based on cameras, wearable devices or RFIDs have various limitations. Camera-based systems are sensitive to lighting conditions and may introduce privacy issues [2]. Wearable devices attached to the body might cause discomfort during exercises [3,4,5]. Besides, some studies have shown decreased adherence to wearable devices over time [6]. RFIDs have been used to track workouts when attached to dumbbells, which is inapplicable to freehand exercise monitoring [7].

A more promising alternative for fitness assistants is to capture human motion through wireless sensing [8,9,10], e.g., using Wi-Fi and RFID backscatter signals [11,12,13,14]. However, existing wireless sensing approaches exhibit three limits. First, the signal transmitters must transmit periodic sinusoid signals, so the wireless channel has to be dedicated to sensing and is not compatible with any data communication. Second, users must exercise within a specific area (such as the first 8–12 Fresnel zones) between a pair of RF signal transceivers [11,14]. Third, they cannot support multiple users exercising together in one room, due to the mutual interference on signal reflections among users.

To overcome the above limits, we propose a cellular-based freehand exercise assistant system, MobiFit. Since the base stations are widely distributed by commercial cellular operators, the user can use MobiFit anywhere just by placing a cellular signal receiver on the ground. MobiFit traces freehand exercises by monitoring the received cellular signals and provides exercise quality indicators, including repetition number, exercise type and duration for each exercise repetition. MobiFit faces two new challenges: (1) The cellular signals transmitted by the base station are beyond MobiFit’s control, which are interwoven with both control messages and traffic data. MobiFit has to extract the signals affected by exercises through uncontrollable and unpredictable cellular signals. (2) The long signal propagation distance between the base station and the receiver leads to the time-variant propagation paths, which eliminates the applicability of Doppler analysis.

**Figure 1:**
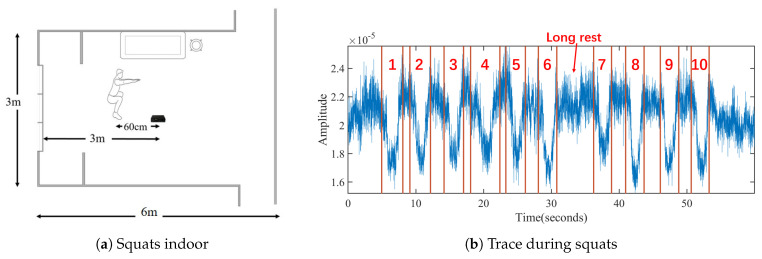
Application scenario of MobiFit and the collected data trace during ten squats with segmentation result [15].

Figure 1 illustrates the typical application scenario (MobiFit Demo: https://youtu.be/Dupmc1LAWTU (accessed on 14 July 2020) and https://www.bilibili.com/video/BV15p4y1S7As/ (accessed on 14 July 2020)), where we conducted the experimental study. We observed that the envelope of the recorded signal sequence fluctuates periodically with the ups and downs of the torso motions during freehand exercises, as shown in Figure 1b. Based on the observation, we construct an analytic model of the received signals and demonstrate the torso block effect on signal propagation as the primary cause for the periodic fluctuations. The analytic model also points out that low-frequency components can help in exercise repetition segmentation and type recognition. Guided by the insights, MobiFit exploits the spectrogram analysis on the received signals and realizes real-time segmentation of exercise repetitions. MobiFit then applies Fast Fourier Transform (FFT) and Discrete Wavelet Transform (DWT) to extract the low-frequency coefficients from each repetition sequence and applies the Support Vector Machine (SVM) for exercise type recognition.

MobiFit tackles the challenges by focusing only on the low-frequency coefficients for repetition counting and type recognition. The frequency band and data rate of cellular signals are high and independent of low-frequency coefficients. Thus, MobiFit does not require an exclusive channel for sensing, solving the first challenge. Since the block effect on signal propagation comes from the torso motion nearby the receiver, the signal propagation path diversity from the base station to the receiver becomes negligible. It not only solves the second challenge but also implies the possibility of providing exercise assistance for multiple users exercising together in the same room.

We implemented the prototype of MobiFit on USRP-1 and RFX900 sub-board to trace the GSM signal and output 100 Hz down-sampled signal sequence for freehand exercise tracking. We deployed MobiFit under three typical scenarios, both indoors and outdoors. We collected 22,960 exercise repetitions of six freehand exercise types performed by ten volunteers over six months. The results confirm that MobiFit counts repetitions with an error ratio as low as 1.4% per exercise session, across all volunteers and exercise types. Overall, 98% of the repetition duration estimation error is within 0.3s.

Moreover, only using the SVM can accurately recognize each exercise repetition with 94.1% accuracy under five-fold cross-validation on all traces. Besides, MobiFit’s recognition is permanent, i.e., robust across two weeks. What is more, MobiFit provides counting and type recognition of every exercise repetition with only 5 s delay, instead of after the whole session required by existing works. Finally, the experiments demonstrate the feasibility of MobiFit supporting multiple users exercising together and exercising both indoors and outdoors.

The main contributions of this work can be summarized as follows:

- This work proposes and verifies the feasibility of applying cellular signals for passive freehand exercise tracking, which sheds light on a new kind of wireless signals for motion sensing, especially at the advent of 5G.
- We propose an analytic model to quantify the impact on the received cellular signals when humans conduct freehand exercise nearby. The analytical model provides two insights for other motion tracking research with cellular signals.
- We propose a real-time freehand exercise repetition segmentation scheme and several low-frequency features for type recognition, which may be further applied in motion repetition counting and recognition with cellular signals.
- We implemented the prototype of MobiFit and evaluated it with extensive experiments, both indoors and outdoors. The results confirm that MobiFit achieves high accuracy in counting and type recognition for freehand exercises.

The rest of the paper is organized as follows. In Section 3, experimental studies are presented to verify the feasibility of MobiFit. In Section 2, we survey the related works and analyze the limitations of existing studies. In Section 4, the analytic model is introduced and used to guide system design. We present the proposed freehand exercise assistant system, MobiFit, in Section 5 and evaluate it in Section 6, followed by the conclusions in Section 7.

## 2. Related Work

We divide and review the existing wireless sensing work into two categories: cellular signal-based and non-cellular signal-based motion sensing systems. The authors of [16,17,18] used the mobile phone as a body-attached cellular signal sensor. Sohn et al. [16] applied the mobile phone to measure and record the surrounding GSM radio environment. Then, they inferred whether the user is walking, driving or staying stationary from the variance of measured signals and counted the step number. Shakra [17] used an Artificial Neural Network to analyze GSM signal strength to estimate whether the user is walking, driving or staying stationary. Anderson et al. [18] also employed the patterns of signal strength fluctuations and changes to the current serving cell and neighboring cells to distinguish between various states of movement such as walking, driving and remaining stationary. Unlike the above research, MobiFit does not require the user to attach their mobile phone to the body during exercises, which is often cumbersome and may cause unwanted motion changes. At the same time, the existing studies based on cellular signals do not count the motions, so it cannot provide a complete exercise monitoring for users.

Recently, LTE-based passive radar [19] achieves moving target detection via Doppler resolution. Chen et al. [20] recognized dynamic hand gesture interaction by analyzing CSI extracted from LTE signals. SpiderMon [21] performs keystroke monitoring using the cellular signals transmitted by commercial base stations. Furthermore, a few 5G prototype systems are proposed and applied to human sensing. For example, Gholampooryazdi et al. [22] enabled crowd-size detection and walking speed recognition. However, these studies only realize motion detection but do not provide motion counting. Thus, they cannot be applied as freehand exercise assistants.

Existing wireless sensing systems with other RF signals can be further classified into dedicated device based, RFID based and WiFi based. For the first category, RF-Capture  [23], RF-Pose  [24] and DFAR [25] use dedicated wireless devices to scan RF reflections, which can recognize and count user’s actions. For the second category, FEMO  [7] attaches passive RFID tags on dumbbells and measures the Doppler shift profile of the backscatter signals to count and recognize repetitions of arm exercises. Motion-Fi  [26] deploys two USRPs to measure the backscattered signal of passive RFID tags to count and recognize free-weight exercises.

WiFi-based motion tracking relies on RSSI or the more detailed CSI to realize activity detection [13,27,28,29,30,31], gesture recognition [32,33,34] and tracking [35,36,37,38,39,40]. WiFit  [41] enables people to practice three kinds of freehand exercises with the body on the line-of-sight between Wi-Fi transceivers. Guo et al. [14] also exploited Wi-Fi to count and recognize repetitions for free-weight exercises. Zhang et al. [42,43,44,45] designed Fresnel zone-based methods to sense and count human activities.

Existing wireless sensing systems study the signal reflection profile by the body, which requires the sender to emit periodic sinusoid signals on the sensing channel. Different from these systems, MobiFit does not take any control of the sender, whose receiver directly makes use of the cellular signals transmitted from the nearby mobile station. Hence, MobiFit does not need a wireless channel exclusively used for sensing.

## 3. Experimental Study

In this section, we first give a brief introduction to GSM signals. Then, we describe the setup, process and observation of the experimental study.

### 3.1. GSM Background

The Global System for Mobile communications (GSM) describes the protocols for cellular networks used by mobile devices such as mobile phones and tablets [46]. GSM uses a cellular network structure to achieve frequency band reuse between cells. To ensure the stability of communication, the size of the cellular is determined by the density of the base station deployed by the operator. GSM only stipulates the maximum cell radius of 35 km. To meet the increasing demand for communication, the base station density is usually at kilometers level. Cellular signal propagation paths are more dynamic than those of Wi-Fi due to the longer distance.

The base station uses both FDMA and TDMA to embed logical channels. The base station uses frequency division multiplexing on the physical frequency bands. Each physical band is further divided into eight time slots for time-sharing, grouped as a TDMA multi-frame. The time slot is the basic unit to embed one logical channel.

**Figure 2:**
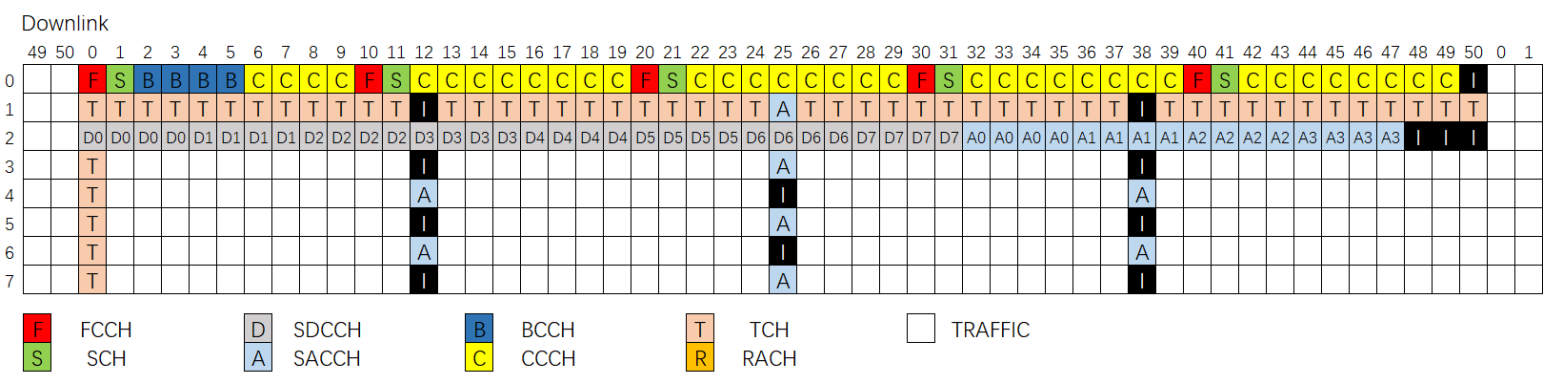
Multi-frame structure on Beacon Channel.

Each base station has a predefined physical frequency band called Beacon Channel, as shown in Figure 2. The control messages and data payloads are interwoven among slots. Beacon Channel loads the control logic channels in Slots 0–2. Slots 3–7 are loaded with data traffic. The base station repeatedly broadcasts TDMA multi-frame over the Beacon Channel.

The mobile terminal obtains the access information of the base stations in a cell by analyzing their corresponding BCCHs. It selects one base station to access according to the signal strength. After the mobile terminal associates with one base station, it keeps monitoring the Beacon Channel for changes in BCCH or controlling messages such as calls to itself. The continuous broadcast feature of Beacon Channel makes it possible for cellular signals to track freehand exercises. However, it also brings two challenges.

### 3.2. Setup

**Figure 3:**
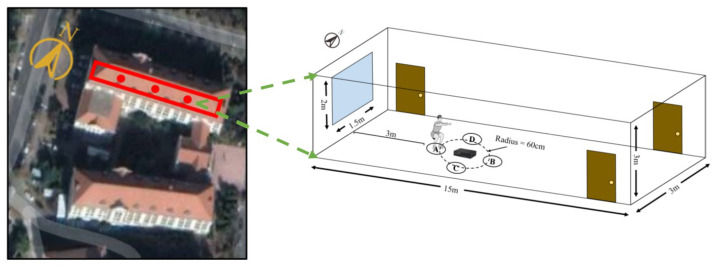
Corridor deployment for experimental study.

To explore the feasibility of using GSM signals to monitor freehand fitness, we conducted experiments in the corridor shown in Figure 3. Since mobile phones do not provide any API to access the cellular signals, the prototype of MobiFit with a USRP-1 and an RFX900 sub-board was used to simulate the whole procedure of receiving GSM signals on the smartphone and output 100 Hz down-sampled sequences for exercise tracking. The receiver continuously received the cellular signal sequence on the Beacon Channel and outputted the signal sequence down-sampled to 100 Hz. The volunteer performed freehand exercises such as squats at a distance of 60 cm around the receiver, which was put on the ground.

**Figure 4:**
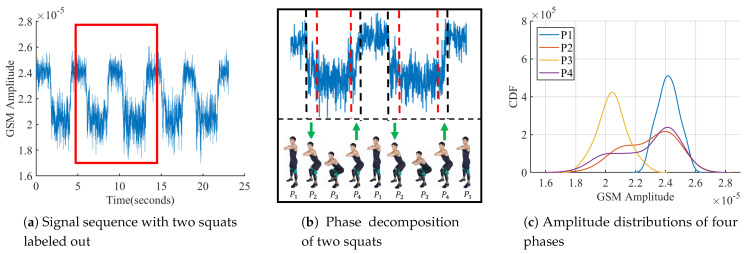
Down-sampled signal sequence while squatting [15].

In the corridor, a volunteer completed ten squats in front of the signal receiver, recorded by video. Figure 4a shows an example of the signal sequence received. Its envelope presents periodic fluctuation. Figure 4b shows a zoom-in view of the signal sequence of the box marked in Figure 4a. The squat exercise can be divided into four phases in sequence: resting, concentric contraction, dropping and eccentric contraction, labeled in Figure 4b as $P_{1}$–$P_{4}$ through video analysis. Every phase of freehand exercises lasts a certain time to train muscle groups. Figure 4b shows that the fluctuation of the signal sequence coincides with the four phases of squat exercises.

The torso moves up and down once during repetition because body weights are the only resistance to work multiple muscle groups in freehand exercises. The periodic torso motion in the consecutive squats will cause periodic impact, which in turn exhibits periodic change on the signal sequence envelope. Thus, the fluctuation period of the sequence envelope will be the same as the exercise repetition period. Figure 4c exhibits the amplitude distributions of the signal sequences in these four phases of squat exercises. Concentric contraction and eccentric contraction have similar distributions in Figure 4c, whose signal sequences are also symmetric with time in Figure 4b. Both resting and dropping phases have normal distributions, but the mean amplitude in the dropping phase is lower than that of resting.

### 3.3. Experiments on Different Positions

To evaluate the effect of the exercise position to the receiver, a volunteer conducted squats on four orthogonal locations of one circle.

**Figure 5:**
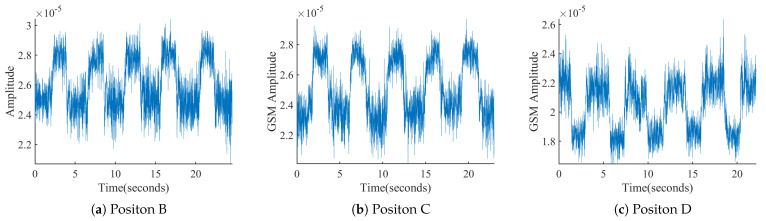
Down-sampled signal sequence recorded while squatting at different positions.

Figure 5 shows the recorded corresponding signal sequences during squatting at Positions B–D. The envelopes of the three sequences also exhibit the phenomenon of ups and downs consistent with the squat. Thus, our observation is extended to: during indoor squatting, the envelope of down-sampled cellular signal sequence shows periodic ups and downs consistent with the squat repetition.

As shown in Figure 4a and Figure 5, all starting parts of the received sequences correspond to the volunteer in the resting phase ($P_{1}$). For squats at Positions A and D, the sequence envelope first falls and then rise back from phase $P_{1}$ to phase $P_{4}$. The sequences obtained at Positions B and C show the opposite process of change.

We queried the actual location of the connected base station, which was located in the northwest direction to the receiver. Thus, the cellular signals propagated from the base station to the receiver in the southeast direction. Thus, the difference between Positions A and D and Positions B and C lies in whether the cellular signals arrive first at the volunteer or the receiver for Positions A and D. Thus, the torso may block a larger portion of the cellular signals from reaching the receiver during the action phase than in the resting phase. This may result in a decrease in the amplitudes of the received signals during phases $P_{2}$–$P_{4}$. For Positions B and C, after some portion of cellular signals passes over the receiver, they will be reflected back to the receiver by the torso during phases $P_{2}$–$P_{4}$. Thus, the sequence envelope will be higher during the action phase than during the resting phase.

### 3.4. Experiments on Different Exercises

**Figure 6:**
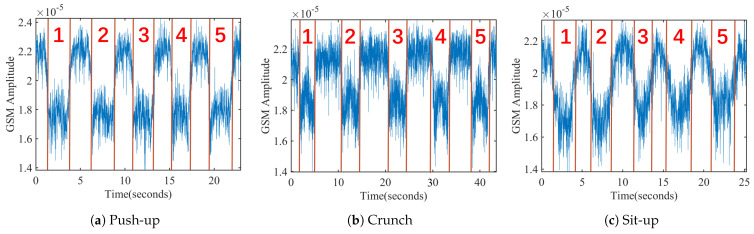
Signal sequences of three different freehand exercises.

We further carried out experiments on other types of freehand exercises, including push-ups, crunches and sit-ups. Figure 6 shows three segments of the recorded signal sequences when a volunteer carries out corresponding exercises at Position A. Since there is also the block effect during the action phases of these three exercises, the sequence envelopes are consistent with the proposed observation.

As a summation, we observed empirically that the envelope of the recorded signal amplitude sequence fluctuates periodically, consistent with the exercise cycle of ups and downs of torso motions during freehand exercises. The observation is confirmed by the proposed analytic model in the next section. The scenario is expanded to outdoor environments in the evaluation section.

## 4. Analytic Model

In this section, we construct the analytic model for the received signals. The analytic model begins with multi-path analysis. Although cellular signals can transverse walls, there are some proportion of signals reflected by the walls and other objects. Hence, cellular signals propagate from the transmitter (base station) to the receiver (mobile terminal) via multiple reflections, especially when the receiver is indoors. The received signals are the superposition of the signals from all the propagation paths.

**Figure 7:**
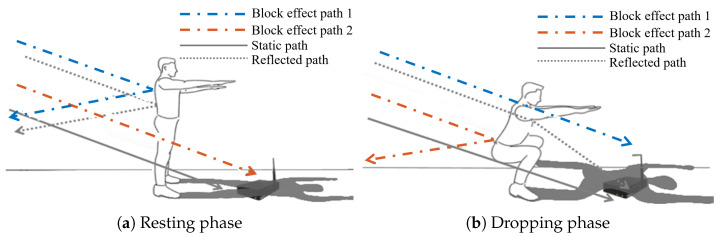
Impact of human motion on signal propagation [15].

As the human body is a good reflector of cellular signals, during freehand exercises, the multi-path propagations of cellular signals arriving at the receiver will be changed according to the torso motion. Figure 7 shows a schematic diagram of cellular signal multi-path propagation under two decomposed phases during squats.

Figure 7a,b corresponds to the resting and dropping phases of squats, respectively. The solid line represents a propagation path unaffected by the torso motion; the dotted line represents the dynamic reflection path created by the torso motion; and the dash-dotted line indicates the block effect on the cellular signal propagation related to the torso motion. Under the resting phase, the cellular signals propagating along the red dash-dotted line can reach the receiver, while the ones propagating along the blue dash-dotted line and the dotted line cannot reach the receiver. Under the dropping phase, the signals propagating along the dotted line are reflected by the torso to reach the receiver. The cellular signals propagating along the blue dash-dotted reach the receiver because there is no blocking. Moreover, the cellular signals propagating along the red dash-dotted line are reflected away from the receiver. In Figure 7, we use the shadow metaphor to describe the variation of the blocking effect of the torso on cellular signal propagation.

To analyze the received signals, we first divided the signal propagation paths based on whether they are affected by human exercise or not, called dynamic and static paths [12,42,47], as shown in Equation (1). $y_{s}\left(f,t\right)$ represents the cellular signal propagating along the static paths not affected by the human motion. $y_{s}\left(f,t\right)$ does not contain the low-frequency components corresponding to our observation, because the payloads are at 10s kbps, and the modulated frequency is over 900 MHz for GSM.

$y_{d}\left(f,t\right)$ represents the superposition of cellular signals propagating along the dynamic paths. $y_{d}\left(f,t\right)$ can be further divided into two terms, as shown in Equation (2). $y_{r}\left(f,t\right)$ represents the superposition of signals reflected by the torso during exercises, shown in Equation (3). $P_{r}$ indicates the path set whose signal propagation distance changes with body motion. $a_{p}\left(f,t\right)$ is the complex-value representation of attenuation along with path *p*. x(t) is the symbol transmitted within time *t*. *c* denotes the speed of light and *f* represents the beacon channel frequency. $d_{p}$ denotes the distance of the path *p*. The reflected path change will cause the frequency or phase shift in $y_{r}\left(f,t\right)$. Wi-Fi- or RFID-based exercise assistants focus on extracting Doppler frequency shift or phase shift for exercise recognition. For MobiFit, the propagation paths of cellular signals are on the kilometer level, much longer than the 10 m level under Wi-Fi or RFID. More reflection exists during cellular signal propagation except for human motion. Thus, $y_{r}\left(f,t\right)$ also does not account for low-frequency fluctuation of the observation.

(1) $$y(f,t)=y_{s}\left(f,t\right)+y_{d}\left(f,t\right)$$

(2) $$y_{d}\left(f,t\right)=y_{r}\left(f,t\right)+y_{b}\left(f,t\right)$$

(3) $$y_{r}\left(f,t\right)=\sum_{p\in p_{r}}a_{p}\left(f,t\right)x\left(t-\frac{d_{p}-\frac{v(t)}{f}}{c}\right)e^{-j2\pi f(t-\frac{d_{p}-\frac{v(t)}{f}}{c})}$$

**Figure 8:**
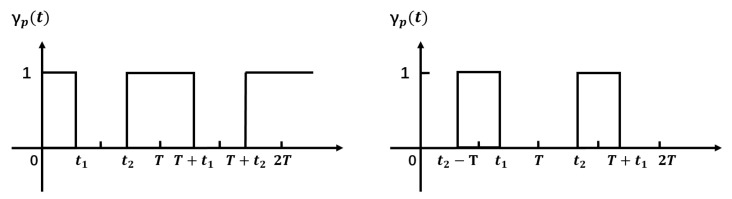
Block effect function of $\gamma_{p}\left(t\right)$ with different duty ratio.

$y_{b}\left(f,t\right)$ represents the superposition of cellular signals related to block effect, calculated in Equation (4). $P_{b}$ indicates the signal propagation path involved in the block effect. Here, the periodic block effect of the torso on signal propagation is represented with a square wave function $\gamma_{p}\left(t\right)$, defined as Equation (5). *T* represents the repetition cycle of freehand fitness; $t_{1}$ and $t_{2}$ mark the start and end timestamp of the block effect on signal propagation along with path *p*. Figure 8 shows two examples of switch functions with different duty cycles.

(4) $$y_{b}\left(f,t\right)=\sum_{p\in p_{b}}\gamma_{p}\left(t\right)a_{p}\left(f,t\right)x\left(t-\frac{d_{p}}{c}\right)e^{-2\pi f(t-\frac{d_{p}}{c})}$$

(5) $$\gamma_{p}\left(t\right)\;=\;\left\{\begin{array}{ccc} 0 & \; & t_{1}\;<\;t\;mod\;T\;<\;t_{2}\\ 1 & \; & otherwise \end{array}\right.$$

The frequency of freehand exercise is quite low (≤1 Hz), referring to experimental observation. $y_{b}\left(f,t\right)$ is the only possible factor which can introduce low-frequency components. First, $\gamma_{p}\left(t\right)$ of all paths share the same cycle *T* of freehand exercise repetition. Second, $y_{b}\left(f,t\right)$ accounts for a large proportion of change of y(f,t) due to the larger area of the torso to block signal propagation. Previous research [26] confirmed that the influence of limbs on wireless signal propagation is far less than that of the torso. Third, the torso’s size is larger than the cellular signal’s wavelength, and the torso is near the receiver. Thus, most of the blocked propagation paths will share similar phases, creating the aggregated fluctuation after superposition. Thus, the switch function $\gamma_{p}\left(t\right)$ leads to the ups and downs of the amplitude envelope of y(f,t). The analytic model explains and verifies the experimental observation, i.e., the torso motion causes periodic ups and downs on the received signal sequence’s envelope with its frequency the same as exercise repetition.

To this end, we summarize the key insights of the analytic model as follows: (1) Each component in $y_{b}\left(f,t\right)$ shares the same cycle *T* of freehand exercise repetitions. By sifting out the low-frequency component near the frequency $T^{-1}$, we may cut out the exercise repetitions from each session. (2) Different kinds of freehand exercises contain different procedures of torso motions, which in turn creates different kinds of block effects. Then, $\gamma_{p}\left(t\right)$ for each involved propagation path will have different duty circle ratio ($\frac{\left|t_{2}-t_{1}\right|}{T}$) for various exercises. Different duty cycle ratio in square wave $\gamma_{p}\left(t\right)$ will create various low-frequency components. These low-frequency components correspond to the characteristic of the torso motion among different types of freehand exercises, which are potential features for exercise type recognition. These two insights form the basis of MobiFit design.

## 5. System Design

**Figure 9:**
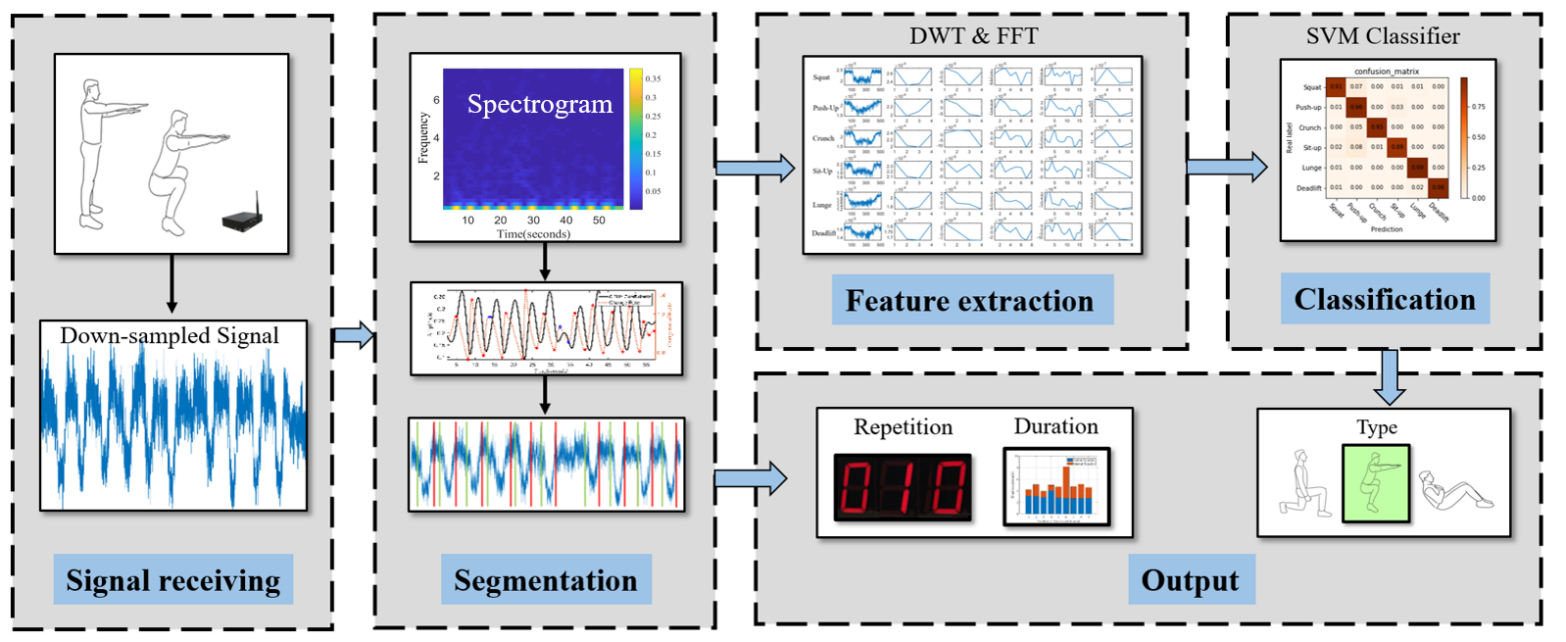
MobiFit framework with an example of a squat session [15].

MobiFit counts and recognizes the exercise repetitions by processing the down-sampled cellular signals. As shown in Figure 9, MobiFit consist of five modules: signal receiving, segmentation, feature extraction, classification and output. The signal receiving module monitors cellular signals and outputs a signal sequence down-sampled to 100 Hz. The segmentation module cuts the signal sequence into exercise repetitions and rest intervals. From the signal sequence of each repetition, the feature extraction module extracts the low-frequency features. Then, the SVM classifier recognizes the type of current repetition. The output module presents the number of repetitions for each exercise type and the duration distribution of exercise repetition and rest intervals. This section focus on the details of the segmentation and feature extraction module.

### 5.1. Segmentation

**Figure 10:**
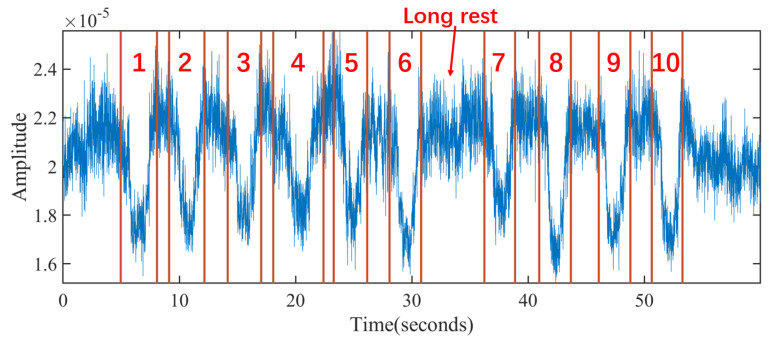
A session of squats performed by a volunteer with red lines and number marking the ground truth of each repetition.

The segmentation module cuts in real-time the down-sampled cellular signal sequence into the sequences of exercise repetitions and rest intervals between two continuous repetitions. The challenge of segmentation lies in the nonuniform rest intervals, which directly limits the applicability of template matching. During exercises, ordinary users cannot control the rest intervals between exercise repetitions due to the lack of fitness ability and experience. Thus, there may be short or long intervals between two repetitions. Figure 10 shows a volunteer’s ten consecutive squats, which has a long rest interval between the sixth and seventh squats.

Inspired by Insight 1 from the analysis model, each exercise repetition has a similar primary frequency corresponding to the exercise repetition cycle. MobiFit performs spectrogram analysis on the cellular signal sequence to study the amplitude changes on the primary frequency for exercise repetition segmentation, which consists of the following three steps shown in Algorithm 1 (Segmentation Code: https://github.com/TGL-Silver/MobiFit (accessed on 26 June 2021)).

**Figure 11:**
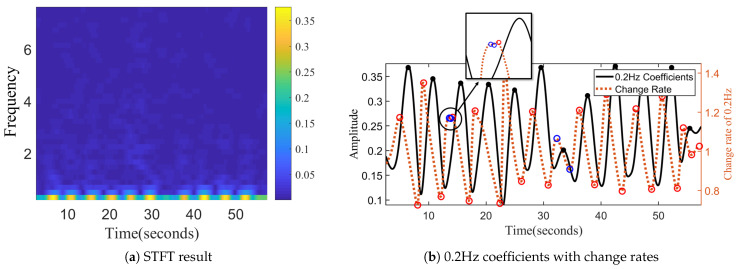
Spectrogram analysis for segmentation [15].

(1) Calculate the amplitude curve corresponding to the primary frequency. MobiFit applies STFT with a sliding window of 512 samples (5.12 s) to cover a single exercise repetition completely in one window. Because most repetitions of the freehand exercise lie within 3–5 s [1], the sliding step is set to 8, corresponding to a time resolution of 0.08 s. Figure 11a shows the spectrogram of the signal sequence in Figure 10. It shows that the highlighted parts of the low-frequency components are consistent with the repetitions. Figure 11b illustrates the amplitude curve of the primary frequency in Figure 11a with a solid black line. Comparing to Figure 10, the peaks on the amplitude curve correspond to the midpoints of each exercise repetition during squats. However, there is an error peak in the long rest interval (32–35 s), which does not correspond to an exercise repetition.

**Algorithm 1:** Segmentation

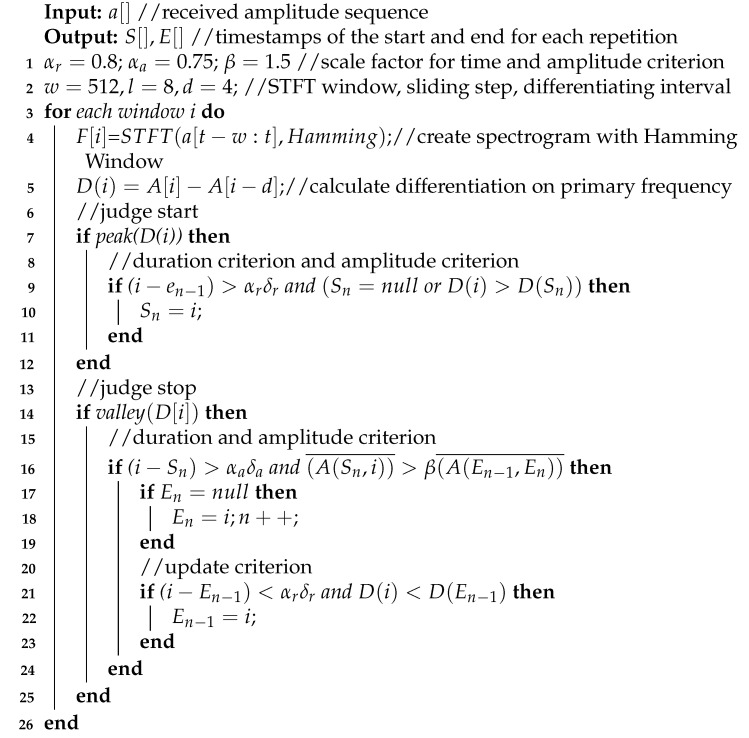



(2) Find the potential timestamps corresponding to the start and end of each exercise repetitions, shown on Lines 7–22. Although the peak of the solid black line in Figure 11b is located near the midpoints of each exercise repetition, MobiFit needs to find the specific timestamps at which each exercise repetition starts and ends. At the beginning of the exercise repetition, the human body changes from the static state to the exercise state, so the amplitude curve of primary frequency changes significantly at this moment. Similarly, at the end of the exercise repetition, the amplitude curve of the primary frequency will also exhibit great change. MobiFit finds the start and end of each repetition by calculating the change rate of the amplitude curve on the primacy frequency, as shown in Equation (6). A(p) is the amplitude of the primary frequency at sliding window *p*, and *d* is the interval for calculating the change rate, set as 4. D(p) is the change rate at sliding window *p*. The red dotted line in Figure 11b shows the change rate of the black line. Each extremum pair from the maximum to the minimum on the change rate curve marks the boundary of one squat repetition in Figure 10, except two extremum pairs around 13 and 33 s, labeled with blue circles. The extremum pair around 13 s may exist due to noise, while some relaxing actions may cause the other pair in the long rest interval. These two extremum pairs do not correspond to the exercise repetition and require further processing.

(6) D(p−d)=A(p)−A(p−d)

(3) Eliminate the false-positive extremum pairs. MobiFit double-checks whether each maximum and minimum are the start or end timestamps of an exercise repetition. For maximum check, the duration criterion is applied to check whether the time length from the end of the last exercise repetition to the current maximum is longer than $\delta_{r}$, which represents the shortest rest interval, shown on Line 9. When multiple consecutive maxima satisfy the duration criterion, MobiFit selects the maxima with the larger value as the start of the current repetition.

MobiFit judges a potential end by the duration and amplitude criteria. The duration criterion for the potential end is that the time length from the start of current repetition to the minimum should be longer than $\delta_{a}$, which represents the shortest repetition duration. If the duration criterion is satisfied, MobiFit then evaluates the amplitude criterion, i.e., the average amplitude of the signal sequence from the start to the minimum should be higher than the average amplitude of previous rest interval, shown on Line 16 in Algorithm 1.

MobiFit applies the end updating criterion when one new minimum appears. Here, the current repetition already has a start and an end. The updating criterion checks whether the current minimum is close to the end of current repetition and its value is smaller, shown on Line 21 in Algorithm 1. If so, MobiFit updates the end of current exercise repetition to this minimum.

Through double-check, MobiFit can eliminate the wrong extremum pairs. We take the process of the ten squats shown in Figure 10 as an example. For the extremum pair around 13 s in Figure 11b, when the first maximum appears, marked with the first blue circle, it satisfies the duration criterion and will be saved as the start of the current repetition. When the minimum of the second blue circle appears, it does not satisfy the duration criterion on end judging and is ignored. When the second maximum with the red circle appears, MobiFit updates the start of current repetition to this maximum because it satisfies the duration criterion and has a larger value. Hence, the extremum pair around 13 s can be eliminated.

For the extremum pair in the rest interval around 32–35 s, the first maximum, marked with a blue circle, satisfies the duration and amplitude criteria to judge the start, which will be saved as the start of current repetition. The minimum around 35 s, marked with a blue circle, satisfies the duration criterion to judge an end but fails the amplitude criterion. Thus, the minimum around 35 s is ignored. Then, the current repetition only labels its start as the maximum around 32 s, while it has no end yet. When the maximum around 37 s appears, it is updated as the start of current repetition because it satisfies the duration criterion, and its value is larger than the maximum at 32 s. Therefore, MobiFit eliminates the error extremum pair around 32–35 s. The elimination process ensures that all the detected extremum pairs represent the starts and ends of exercise repetitions, which realizes the repetition segmentation.

(7) $$N_{est}=size\left(t_{s}\right)$$

(8) $$DA_{est}\left[i\right]=t_{e}\left[i\right]-t_{s}\left[i\right],i=1,...,N_{est}$$

(9) $$DI_{est}\left[i\right]=t_{s}\left[i\right]-t_{e}\left[i-1\right],i=2,...,N_{est}$$

After segmentation, MobiFit records two arrays $t_{s}$ and $t_{e}$, which store the start and end of each repetition, respectively. Based on these two arrays, MobiFit calculates three exercise indicators using Equations (7)–(9). $N_{est}$ represents the repetition number of one exercise session, which evaluates whether one session satisfies the fitness requirement of the repetition number. $DA_{est}$ and $DI_{est}$ indicate the duration of each exercise repetition and rest interval, respectively. The distribution of $DA_{est}$ shows the stability of each repetition and whether the duration of each repetition satisfies the time length requirement; the distribution of $DI_{est}$ shows the stability of rest intervals.

**Figure 12:**
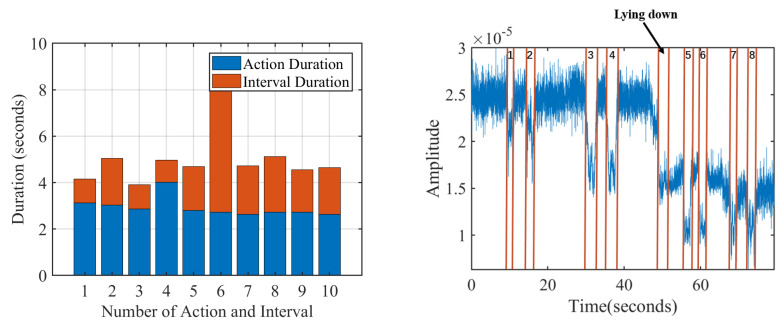
Repetition analysis results of MobiFit [15].

For the 10 squats in Figure 10, Figure 12a shows the duration distribution of each squat repetition and rest interval after segmentation. The ten squats were all completed in 2–4 s, reaching the fitness standard. However, there is one long rest interval after the sixth repetitions, reminding the user to control the rest interval in future training.

One further question for segmentation is that there may be exercise type switching inside one session, where extra actions may cause counting errors. For example, Figure 12b shows that volunteer $v_{1}$ tries two consecutive deficit deadlifts, two squats, two sit-ups and two crunches in one session. The red lines and numbers show the segmentation results of MobiFit, which correctly cut out eight repetitions. However, a false positive repetition appears inside the process of changing from the standing posture to the lying posture. For reducing false positives, existing studies [7,26] require that each session should contain at least three consecutive repetitions of one type to distinguish between exercise and non-exercise actions. MobiFit revises this rule to eliminate non-exercise repetition, whose type is different from previous or subsequent repetitions, called segmentation refinement.

### 5.2. Feature Extraction

From the signal sequence of each exercise repetition, MobiFit extracts the low-frequency features representing different types of exercises according to Insight 2 from the analytic model. (1) Insight 2 illustrates that different freehand exercise may have different $\gamma_{p}\left(t\right)$ of the human block effect during exercising. (2) We apply two frequency analysis tools, FFT and DWT, to calculate the low-frequency features from the repetition traces. Before frequency analysis, MobiFit resamples every repetition to 512 using Cubic Spline Interpolation.

FFT decomposes a signal into a frequency spectrum. Because the major frequency of freehand exercises lie in the range of 0.2–1 Hz, we take the normalized coefficients from the second to the fifth lowest frequencies. This frequency range corresponds to 0.4–1.0 Hz. We omit the basic frequency of 0.2 Hz, because all exercise types share the same cycle, which is used in segmentation.

**Figure 13:**
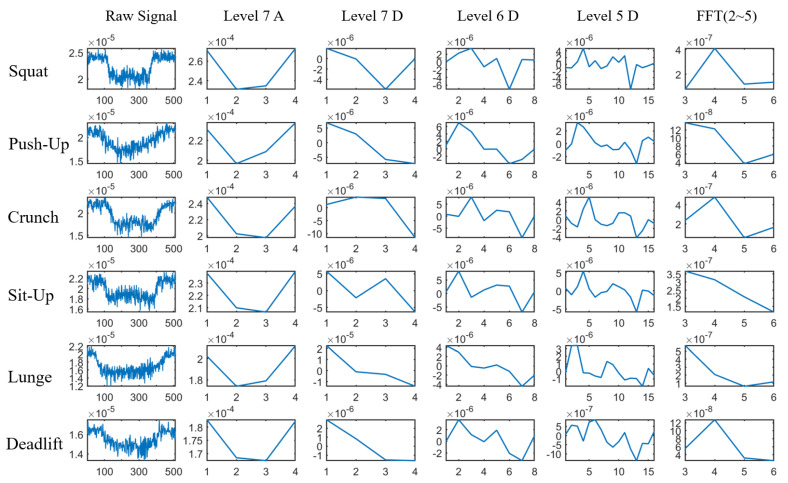
Features extracted from repetitions of six exercise types.

Since FFT loses time resolution, MobiFit applies DWT to measure the low-frequency components with specific time resolution. Different exercises may have different frequency components in the start, middle and end phase of an exercise repetition. For example, sit-ups have more low-frequency components than crunches in the middle phase, as shown in Figure 6. MobiFit decomposes the signal sequence into seven levels, using the low-frequency coefficients of the seventh level and the high-frequency coefficients of the fifth to seventh levels as features. The frequency ranges of these four groups of wavelet coefficients are 0–0.78, 0.78–1.56, 1.56–3.13 and 3.13–6.25 Hz. Figure 13 shows the signal sequences and corresponding features of six exercise types, which exhibit some differences among these features for various types.

Note that MobiFit cut out one repetition only with a delay of one sliding window. The feature extraction is carried out immediately after segmentation, and then the SVM classifier is called to recognize the exercise type of current repetition. The features used in SVM are the second to fifth low-frequency coefficients extracted by FFT and the low-frequency coefficients of the seventh and high-frequency coefficients of the fifth to seventh levels extract by DWT. MobiFit realizes real-time repetition counting and recognition.

## 6. Evaluation

In this section, we present the implementation, deployment and experiment process. Then, we evaluate MobiFit’s counting error, recognition accuracy and duration estimation of exercise repetition and rest intervals, comparing to Motion-Fi [26]. We also evaluate the feasibility of using MobiFit for multiple people exercising together and outdoors.

### 6.1. Setting and Process

**Figure 14:**
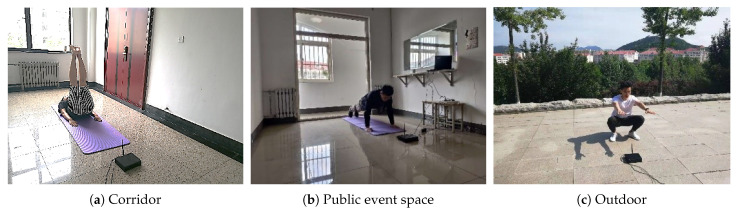
Evaluation scenarios [15].

We conducted experiments in a corridor, a public room and the observation deck on campus, as shown in Figure 14. Our experimental studies show that the envelope of the received signal sequences exhibit the same periodic ups and downs when a volunteer squats at four different locations nearby. Besides, as we do not require the users to know the position of the cellular base station. The volunteer conducted freehand exercises at the different locations in the room for a long period (over six months). The key to segment and classify each exercise repetition lies in the analysis of low-frequency features from the received signal sequence. Because the low-frequency features are not affected by the locations where volunteers exercise, the proposed system can count and recognize repetitions for different exercises. The only requirement on deployment is that the receiver should be put on the ground and the user exercise at a position about 60 cm away from the receiver.

**Figure 15:**
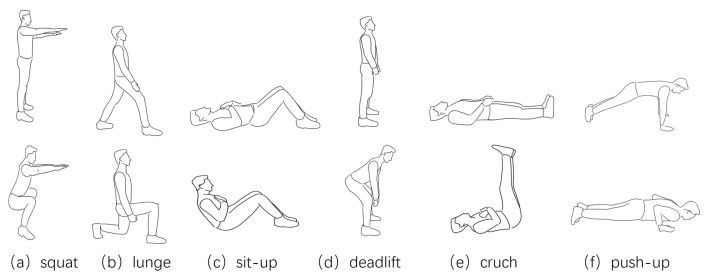
Six kinds of freehand exercises [15].

Ten volunteers were recruited to participate in the experiments, whose age and body parameters are shown in Table 1. In general, the volunteers recruited have low BMIs, i.e., they are thin. MobiFit counts and recognizes exercise repetitions majorly through analyzing the block effect of the torso on cellular signal propagation, so thin bodies increase the difficulty for MobiFit. There are no restrictions on clothes for volunteers. After discussing with ten volunteers on exercise difficulty and quality, we chose six kinds of freehand exercises: squats, lunges, sit-ups, deadlifts, crunches and push-ups (Figure 15). Considering upper limb strength, the females conducted knee push-ups. We hired a fitness instructor to explain and demonstrate the motion mechanism for each type of freehand exercise and guide the volunteers during the experiments.

In order to ensure fitness improvement and avoid possible physical injury, we set the minimum requirement of 10 repetitions for an exercise session. The volunteer was allowed to increase the repetition number in one session based on her/his physical condition. An exercise session could include different kinds of freehand exercises. In the first two weeks, we required the volunteers to complete at least one session for each kind of freehand exercises per day. After two weeks, volunteers exercised in the corridor or public space according to their own exercise frequency. After each session, the volunteers recorded the meta-data, including name, exercise type and repetition number. The whole experiment lasted over six months. The final session and repetition numbers for each volunteer are also shown in Table 1. All volunteers completed 22,960 repetitions. To evaluate the duration accuracy of each exercise repetition and rest interval, we recorded the first 100 squats with cameras for each volunteer.

### 6.2. Results

Existing studies except for Motion-Fi [26] exploit MIMO information from the receiving signals, while MobiFit only monitors single cellular band. Hence, we compared the results with Motion-Fi. Note that Motion-Fi cannot distinguish between exercise repetitions and rest intervals, which are all combined as exercise repetitions.

#### 6.2.1. Repetition Counting

**Figure 16:**
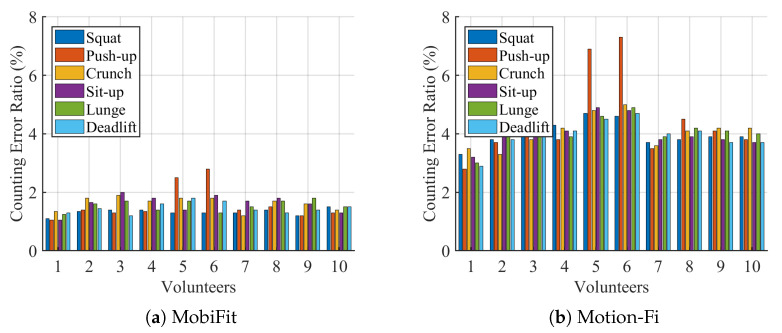
Counting error ratio comparison.

**Figure 17:**
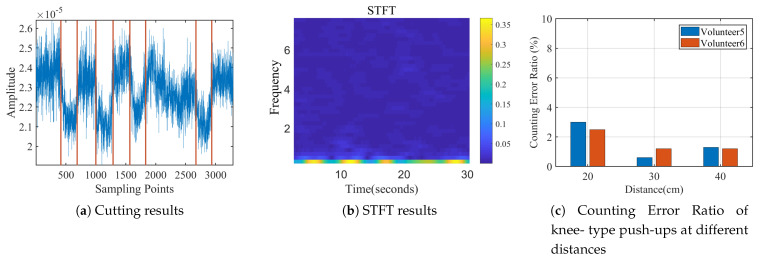
Experiment about knee-type push-ups conducted by volunteers.

For all the traces collected indoors, Figure 16 shows the comparison on counting error ratio between MobiFit and Motion-Fi for every exercise type and each volunteer. Here, the counting error ratio is defined in Equation (10). $N_{est}$ is the estimated repetition number for one session and $N_{truth}$ is the repetition number recorded by a volunteer. The counting error ratio of MobiFit is the result without applying the refinement from exercise type recognition. It shows that most counting error ratio of MobiFit is less than 2%, except for push-ups practiced by two females ($v_{5}$ and $v_{6}$). We show one typical error session of $v_{5}$ in Figure 17 with its corresponding spectrogram. There is one missed repetition around Sample 2250. There are two factors that cause the counting miss. First, Volunteers $v_{5}$ and $v_{6}$ are both petite females, whose torso is relatively small. Second, they practiced knee push-ups, whose motion is also lower comparing to push-ups done by male volunteers. For this particular situation, a closer distance between the petite females and the receiver would decrease counting error, which introduces more variation on the block effect. $v_{5}$ and $v_{6}$ further conducted knee push-ups at 40 cm to the receiver, whose counting error ratios are 0.6% and 1.3%, respectively, as shown in Figure 17c.

(10) $$Counting\;Error\;Ratio=|\frac{N_{est}-N_{truth}}{N_{truth}}|\times 100\%$$

**Figure 18:**
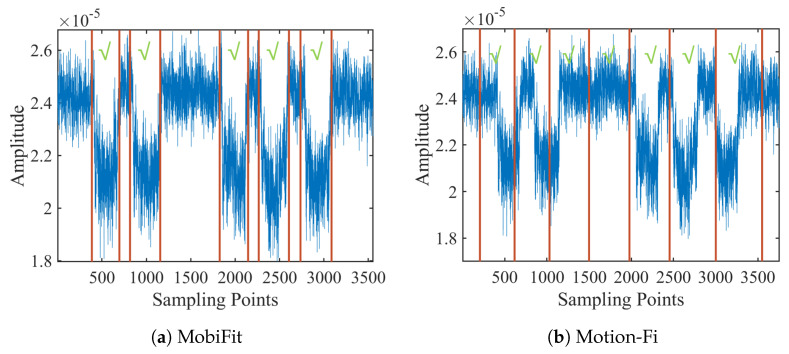
Counting comparison with checks labeled repetitions.

Figure 16b shows the counting error ratio of Motion-Fi on the same traces. Except for the count error ratio of 3% of $v_{1}$, all the other volunteers bear the counting error ratio of over 4%. Motion-Fi uses a template-matching approach for repetition segmentation, requiring the repetition to be stable in a session. However, it is difficult for users who lack exercise experience to keep the workout stable, especially with rest intervals. Figure 18 compares segmentation using MobiFit and Motion-Fi for a squat trace performed by $v_{6}$. MobiFit correctly segments the long rest interval between the second and third squats. However, Motion-Fi does not distinguish the exercise repetition and rest interval, which wrongly counts the long rest interval as two repetitions, as shown in Figure 18b.

**Figure 19:**
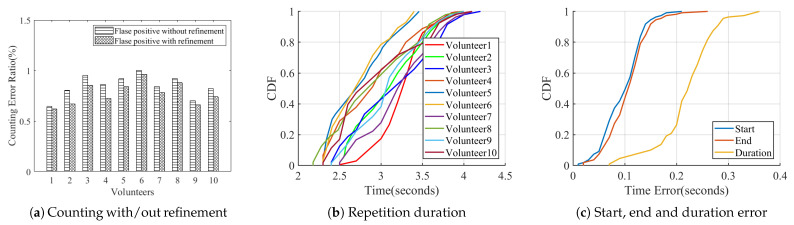
Segmentation results [15].

When volunteers repeat exercise repetitions at least twice for each type, MobiFit uses type recognition to reduce the counting error ratio. Figure 19a compares the false positive rate of counting with/without the refinement. The results are improved after applying refinement, which removes the non-exercise actions during exercise type switching. Because most wrongly counted actions come from switching between standing and lying postures, they do not happen repeatedly. After refinement, the average counting error ratio is reduced to 1.4%.

After segmentation, MobiFit also outputs the duration of each exercise repetition and rest interval. The duration distribution shows the stability of volunteers during freehand exercises, which is an indicator of the quality of freehand exercises. Figure 19b shows the cumulative distribution function (CDF) of repetition duration on squats for ten volunteers. Volunteer $v_{1}$ exhibits the most stable repetition duration, with over 65% of their squats being between 3 and 3.5 s. Figure 19b also shows that female Volunteers $v_{5}$ and $v_{6}$ have the shortest duration, which needs to be prolonged to achieve the exercise effect of squats.

To evaluate accuracy on the duration estimation, we recorded the first 100 squats for each volunteer. The video rate is 30 frames per second. We manually analyzed the video and selected the start and end frames for each squat, recording the corresponding timestamp as $TS_{truth}$ and $TE_{truth}$. We calculated the start, end and duration error for each squat repetition, using Equations (11)–(13), respectively. Figure 19c shows the CDF of estimation error on start, end and duration. The start and end errors are within 0.21 and 0.27 s, respectively. Overall, 98% of the duration error is within 0.3 s.

(11) $$Start\;Time\;Error=|TS_{est}-TS_{truth}|$$

(12) $$End\;Time\;Error=|TE_{est}-TE_{truth}|$$

(13) $$Duration\;Error=|\left(TE_{est}-TS_{est}\right)-\left(TE_{truth}-TS_{truth}\right)|$$

#### 6.2.2. Recognition Classification

We evaluated the recognition accuracy of MobiFit with two procedures and compared the results of Motion-Fi. The first took all traces for five-fold cross-validation and then selected the best combination of features and classifiers. The second used the traces of the first week to train the model. Then, the recognition accuracy was evaluated day by day with the traces in the second week and after three months to show recognition permanence, i.e., the recognition accuracy does not decline quickly with time.

**Cross-validationon Features and classifier:** For feature combinations, we exploited the classifier of Cubic SVM, whose results are shown in Table 2, compared with Motion-Fi. The first two rows indicate that the low-frequency features outperform the time domain features of Motion-Fi, which confirms Insight 2 in the analytic model. After adding the FFT coefficient, the mean recognition accuracy across all volunteers is 94.1%. There are two reasons Motion-Fi’s is inferior to MobiFit. First, Motion-Fi does not distinguish the exercise repetition and rest interval, increasing the difficulty of recognition. Second, all the features used in Motion-Fi are time-domain features, which are affected by the variance of payload traffic contained in cellular signals.

**Figure 20:**
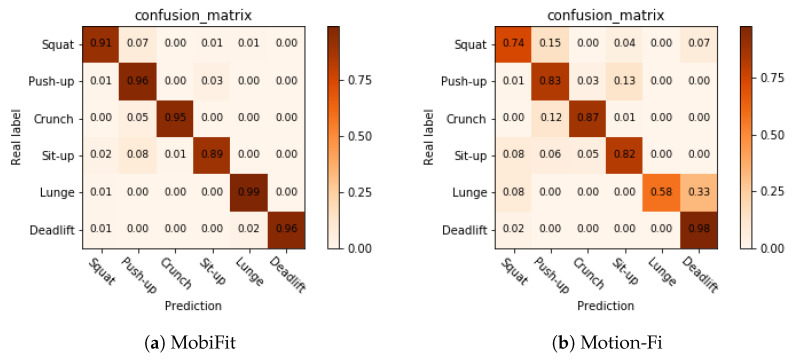
Comparison on confusion matrix of type recognition for Volunteer $v_{3}$.

We further dug into the confusion matrix of $v_{3}$, as shown in Figure 20. Motion-Fi misclassifies 33% of lunges to deadlifts, and its classification accuracy of sit-ups is only 82%. On the contrary, MobiFit achieves high classification accuracy on lunges. For MobiFit, the highest classification error is misclassifying 8% of sit-ups as push-ups, as sit-ups are harder to complete stably.

With the best feature combination, we evaluated recognition accuracy on different classifiers, as shown in Table 3. The table shows that all the classifiers with high-order kernels achieve above 90% accuracies, reflecting that the characteristics of low-frequency features are in high orders. The best classifier is the SVM with the cubic kernel. Therefore, MobiFit chooses the low-frequency features from FFT and DWT and applies the Cubic SVM as the classifier.

**Permanence:** To verify the permanence of MobiFit on recognition, we used the first-week traces to train the classifier. The data traces in the second week were used to test the accuracy day by day, and we further used the data traces after three months for verification. We did the same permanence evaluation with Motion-Fi. Table 4 shows the permanence results across all volunteers. MobiFit’s classification accuracy decreases slowly over time, remaining above 92% within one week and dropping to 81% after three months. The drop after three months may come from the seasonal clothing changes that cause action distortion during exercises. Motion-Fi’s classification accuracy decays more quickly over time than that of MobiFit.

We evaluated and compared the performance of MobiFit and Motion-Fi in terms of counting accuracy, activity recognition accuracy and permanence in the evaluation section. Through comparison, we first confirm that the proposed repetition segmentation scheme outperforms the template matching algorithm of Motion-Fi on the accuracy of repetition counting. Second, we show the proposed low-frequency features are better than time-domain features extracted by Motion-Fi for type classification.

### 6.3. Parameter Evaluation

In this section, we evaluate the deployment parameters, including the distance between the receiver and the volunteer, the deployment height of the receiver and the sliding window size of spectrogram analysis. Finally, we evaluate the feasibility of using MobiFit outdoors and for multiple people exercising together.

**Figure 21:**
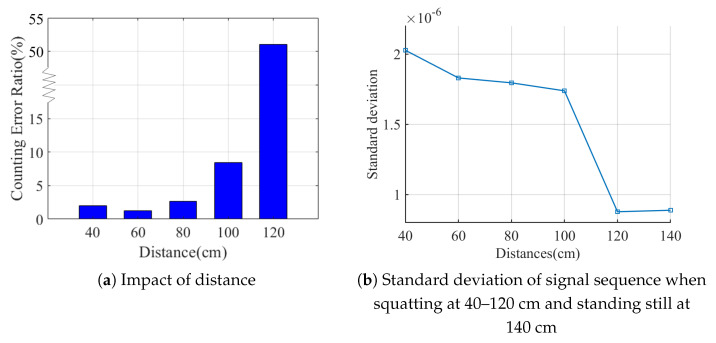
Impact of different distances and heights.

The distance between user and receiver affects the block effect of the torso on signal propagation. The receiver will fall to capture the block effect due to large distances. We studied the impact of different distances between user and receiver on repetition counting. On account of the stability during freehand exercises, we selected Volunteer $v_{1}$ to carry out squats at 40, 60, 80, 100 and 120 cm away from the receiver. Figure 21a shows that MobiFit achieves counting error ratio below 3% within 80 cm. When the distance reaches 120 cm, the counting error is over 50%. It reveals that the user should exercise at a distance of 40–80 cm from the receiver. During the deployment of MobiFit, the distance of 60 cm is taken as default. It also suggests the possibility to support multiple people exercising together when they are more than 120 cm apart.

To further explore the block effect beyond 120 cm, we collected the signal sequence when $v_{1}$ stands stationary at 140 cm for about 50 s. Figure 21b compares the signal sequences’ standard deviations when $v_{1}$ squats at five positions and stands at 140 cm. The standard deviation of signal sequence when squatting at 120 cm is close to that of the static sequence at 140 cm, which confirms that the receiver cannot capture freehand exercises beyond 120 cm.

**Figure 22:**
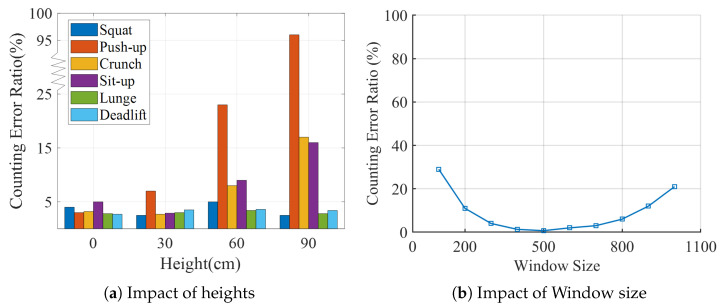
Impact of different deployment heights and window sizes.

When volunteers perform different freehand exercises, their torsos experience different heights during workouts, so we evaluated the impact of different heights to put the receiver on counting error. We placed the receiver at 0, 30, 60 and 90 cm above the ground. $v_{1}$ carries out six types of freehand exercises for each receiver’s height. Figure 22a shows the counting error ratio. Push-ups are mostly affected by different heights, because the torso is close to the ground when doing push-ups, undoubtedly limiting the variation of block effect for high receiver height. For the receiver’s height of 60 cm, the crunch and sit-up errors also increase clearly, as the highest point of the torso is no more than 100 cm for most people during these two workouts. The squat counting error is relatively small because the lowest point of the torso is at least 40–50 cm. In general, the recommended deployment of MobiFit is to place the receiver on the ground, taking a step back and practicing in front of the receiver for most freehand exercises.

The spectrogram analysis uses a sliding window; therefore, the choice of the window size is critical to the counting result. Since MobiFit only deals with 100 Hz down-sampled signals, we cannot observe the frequency components below 1 Hz when the window length is smaller than 100. On the contrary, when the window is too large, such as 1000, a window may contain more than one repetition because the exercise repetition is usually within 3–5 s. To evaluate the impact of STFT’s window size on counting, we increased the window size from 100 to 1000 with steps of 100. We used Volunteer $v_{1}$’s 100 squats for evaluation. Figure 22b depicts the minimum count ratio achieves at window size 500. Since the window size is recommended to be a power of 2 when performing DWT, MobiFit selects the window size as 512.

**Figure 23:**
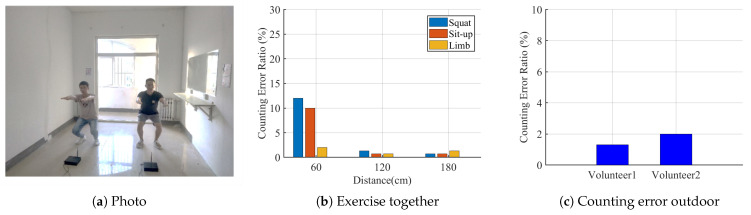
Results of exercising together and outdoors [15].

**Exercising together:**Figure 23a shows the photo when $v_{3}$ and $v_{4}$ are squatting together. Both volunteers work out in front of their corresponding receivers. We set three distances between two volunteers at 60, 120 and 180 cm. $v_{3}$ only carried out squats, while $v_{4}$ conducted three types of activities, squat, sit-up and limb exercises, such as raising arms or kicks. The counting errors of $v_{3}$ are shown in Figure 23b. When the two volunteers are 60 cm apart, the squats and sit-ups of Volunteer $v_{4}$ has more effect on the counting error of $v_{3}$, compared to when $v_{4}$ was conducting limb exercises. Here, $v_{4}$ was about 85cm from $v_{3}$’s receiver, which is still not far enough to avoid his interference. It also reveals that the torso has a more significant impact on cellular signal propagation than limbs. When the two volunteers were 120 cm apart, the count error of $v_{3}$ is below 2%. It confirms that MobiFit supports multiple users exercising together, if they are 120 cm apart.

**Outdoor:**$v_{1}$ and $v_{2}$ conducted squats on the observation deck on campus, as shown in Figure 14c. $v_{1}$ and $v_{2}$ exercised in front of their receiver and collected 150 repetitions each. Figure 23 shows the counting error ratio of 1.3% and 2% for $v_{1}$ and $v_{2}$, respectively, consistent with the results indoors, which confirms the feasibility of using MobiFit outdoors.

## 7. Conclusions

In this paper, we study the feasibility of using ubiquitous cellular signals to help users perform effective freehand exercises. MobiFit works in a contactless manner, which can automatically count and recognize six freehand exercises and provide exercise repetition and rest interval distribution for each session to evaluate workout quality. To build MobiFit, we study the relationship between freehand exercises and received down-sample cellular signal sequence, formulate the torso block effect on cellular signal propagation and construct the analytic signal model. Moreover, we propose a spectrogram analysis method to segment the exercise repetition and rest interval of each repetition and count the repetition number. Based on the segmented phases of each repetition, we extract low-frequency features to classify different types of freehand exercises. MobiFit achieves an accuracy of 98.6% in repetition counting, 94.1% in exercise classification and low repetition duration estimating error within 0.3 s. Moreover, the experiments confirm the feasibility of MobiFit both indoors and outdoors and to support multiple users exercising together.

## Figures and Tables

**Table 1:** Detailed parameters for ten volunteers [15].

Volunteer	Gender	Height	Weight	BMI	Age	Session	Repetition
$v_{1}$	male	184	72	21.3	24	249	2670
$v_{2}$	male	180	75	23.1	23	224	2307
$v_{3}$	male	177	70	22.3	22	216	2246
$v_{4}$	male	175	67	21.9	23	222	2375
$v_{5}$	famale	166	55	20.0	24	192	1971
$v_{6}$	famale	165	50	18.4	22	195	1988
$v_{7}$	male	170	65	22.5	26	222	2298
$v_{8}$	male	173	70	23.4	25	219	2276
$v_{9}$	male	176	78	25.2	23	230	2532
$v_{10}$	male	178	85	26.8	24	217	2297

**Table 2:** Recognition comparison of MobiFit, Motion-Fi [15].

Features Volunteer	1	2	3	4	5	6	7	8	9	10	Mean
Motion-Fi	83.5%	80.2%	81.4%	84.2%	82.9%	82.1%	82.6%	80.7%	81.3%	83.6%	82.3%
Wavelt	92.4%	91.3%	87.3%	91.7%	91.4%	90.7%	91.4%	90.6%	91.5%	91.8%	91%
Wavelt+FFT	94.3%	93.4%	95.2%	94.6%	94.2%	95.2%	95.3%	92.7%	93.6%	92.4%	94.1%

**Table 3:** Classification comparison of classifiers of MobiFit

Method	Tree	Ensemble	KNN	SVM:Cubic	Linear	Quadratic	Gaussian
Accuracy	80.6%	89.7%	82.4%	**94.1**%	88.6%	91.4%	90.2%

**Table 4:** Classification permanence of MobiFit, Motion-Fi [15].

Day	Day 1	Day 2	Day 3	Day 4	Day 5	Day 6	Day 7	After 3 Months
MobiFit(%)	96.5	95.9	95.2	94.7	93.6	93.4	92.7	81
Motion-Fi(%)	93.4	90.3	87.2	85.4	80.6	82.4	80.2	43.5

## Data Availability

Not applicable.

## References

[1] Sharma A. (2012). Fitness on the Go: The Anytime Anywhere Holistic Workout for Busy People.

[2] Zhao W., Feng H., Lun R., Espy D.D., Reinthal M.A. A Kinect-based rehabilitation exercise monitoring and guidance system. Proceedings of the 2014 IEEE 5th International Conference on Software Engineering and Service Science.

[3] Hao T., Xing G., Zhou G. (2015). RunBuddy: A Smartphone System for Running Rhythm Monitoring. Proceedings of the 2015 ACM International Joint Conference on Pervasive and Ubiquitous Computing.

[4] Guo X., Liu J., Chen Y. FitCoach: Virtual fitness coach empowered by wearable mobile devices. Proceedings of the IEEE INFOCOM 2017—IEEE Conference on Computer Communications.

[5] Bian S., Rey V.F., Hevesi P., Lukowicz P. Passive Capacitive based Approach for Full Body Gym Workout Recognition and Counting. Proceedings of the 2019 IEEE International Conference on Pervasive Computing and Communications (PerCom).

[6] Lazar A., Koehler C., Tanenbaum J., Nguyen D.H. (2015). Why We Use and Abandon Smart Devices. Proceedings of the 2015 ACM International Joint Conference on Pervasive and Ubiquitous Computing.

[7] Ding H., Han J., Shangguan L., Xi W., Jiang Z., Yang Z., Zhou Z., Yang P., Zhao J. (2017). A platform for free-weight exercise monitoring with passive tags. IEEE Trans. Mob. Comput..

[8] Shukri S., Kamarudin L.M. (2017). Device free localization technology for human detection and counting with RF sensor networks: A review. J. Netw. Comput. Appl..

[9] Ma Y., Zhou G., Wang S. (2019). WiFi Sensing with Channel State Information: A Survey. ACM Comput. Surv..

[10] Liu J., Teng G., Hong F. (2020). Human Activity Sensing with Wireless Signals: A Survey. Sensors.

[11] Hristov H.D. (2000). Fresnel Zones in Wireless Links, Zone Plate Lenses and Antennas.

[12] Wang W., Liu A.X., Shahzad M., Ling K., Lu S. Understanding and Modeling of WiFi Signal Based Human Activity Recognition. Proceedings of the 21st Annual International Conference on Mobile Computing and Networking.

[13] Wang H., Zhang D., Wang Y., Ma J., Wang Y., Li S. (2016). RT-Fall: A Real-time and Contactless Fall Detection System with Commodity WiFi Devices. IEEE Trans. Mob. Comput..

[14] Guo X., Liu J., Shi C., Liu H., Chen Y., Chuah M.C. (2018). Device-free Personalized Fitness Assistant Using WiFi. ACM on Interactive, Mobile, Wearable and Ubiquitous Technologies. Proc. ACM Interact. Mob. Wearable Ubiquitous Technol..

[15] Teng G., Xu Y., Hong F., Qi J., Jiang R., Liu C., Guo Z. MobiFit: Contactless Fitness Assistant for Freehand Exercises Using Just One Cellular Signal Receiver. Proceedings of the 16th International Conference on Mobility, Sensing and Networking.

[16] Sohn T., Varshavsky A., LaMarca A., Chen M.Y., Choudhury T., Smith I., Consolvo S., Hightower J., Griswold W.G., de Lara E., Dourish P., Friday A. (2006). Mobility Detection Using Everyday GSM Traces. UbiComp 2006: Ubiquitous Computing.

[17] Anderson I., Maitland J., Sherwood S., Barkhuus L., Chalmers M., Hall M., Brown B., Muller H. (2007). Shakra: Tracking and Sharing Daily Activity Levels with Unaugmented Mobile Phones. Mob. Netw. Appl..

[18] Anderson I., Muller H. (2006). Practical Activity Recognition using GSM Data.

[19] Abdullah R.S.A.R., Salah A., Rashid N. (2015). Moving target detection by using new LTE-based passive radar. Prog. Electromagn. Res. B.

[20] Chen W., Niu K., Zhao D., Zheng R., Wu D., Wang W., Wang L., Zhang D. Robust Dynamic Hand Gesture Interaction using LTE Terminals. Proceedings of the 2020 19th ACM/IEEE International Conference on Information Processing in Sensor Networks (IPSN).

[21] Ling K., Liu Y., Sun K., Wang W., Xie L., Gu Q. SpiderMon: Towards Using Cell Towers as Illuminating Sources for Keystroke Monitoring. Proceedings of the IEEE INFOCOM 2020—IEEE Conference on Computer Communications.

[22] Gholampooryazdi B., Singh I., Sigg S. 5G Ubiquitous Sensing: Passive Environmental Perception in Cellular Systems. Proceedings of the 2017 IEEE Vehicular Technology Conference (VTC-Fall).

[23] Adib F., Hsu C.Y., Mao H., Katabi D., Durand F. (2015). Capturing the human figure through a wall. ACM Trans. Graph. (TOG).

[24] Zhao M., Li T., Abu Alsheikh M., Tian Y., Zhao H., Torralba A., Katabi D. Through-wall human pose estimation using radio signals. Proceedings of the IEEE Conference on Computer Vision and Pattern Recognition.

[25] Sigg S., Scholz M., Shi S., Ji Y., Beigl M. (2014). RF-Sensing of Activities from Non-Cooperative Subjects in Device-Free Recognition Systems Using Ambient and Local Signals. IEEE Trans. Mob. Comput..

[26] Xiao N., Yang P., Yan Y., Zhou H., Li X.Y. Motion-Fi: Recognizing and Counting Repetitive Motions with Passive Wireless Backscattering. Proceedings of the IEEE INFOCOM 2018-IEEE Conference on Computer Communications.

[27] Han C., Wu K., Wang Y., Ni L.M. WiFall: Device-Free Fall Detection by Wireless Networks. Proceedings of the IEEE INFOCOM 2014—IEEE Conference on Computer Communications.

[28] Zheng X., Wang J., Shangguan L., Zhou Z., Liu Y. Smokey: Ubiquitous smoking detection with commercial wifi infrastructures. Proceedings of the IEEE INFOCOM 2016—The 35th Annual IEEE International Conference on Computer Communications.

[29] Arshad S., Feng C., Liu Y., Hu Y., Yu R., Zhou S., Li H. Wi-chase: A WiFi based human activity recognition system for sensorless environments. Proceedings of the 2017 IEEE 18th International Symposium on A World of Wireless, Mobile and Multimedia Networks (WoWMoM).

[30] Wang Y., Liu J., Chen Y., Gruteser M., Yang J., Liu H. E-eyes: device-free location-oriented activity identification using fine-grained wifi signatures. Proceedings of the 20th Annual International Conference on Mobile Computing and Networking.

[31] Dong Z., Li F., Ying J., Pahlavan K. (2018). Indoor Motion Detection Using Wi-Fi Channel State Information in Flat Floor Environments Versus in Staircase Environments. Sensors.

[32] He W., Wu K., Zou Y., Ming Z. Wig: Wifi-based gesture recognition system. Proceedings of the 2015 24th International Conference on Computer Communication and Networks (ICCCN).

[33] Zhou Q., Xing J., Li J., Yang Q. A device-free number gesture recognition approach based on deep learning. Proceedings of the 2016 12th International Conference on Computational Intelligence and Security (CIS).

[34] Venkatnarayan R.H., Page G., Shahzad M. Multi-user gesture recognition using WiFi. Proceedings of the 16th Annual International Conference on Mobile Systems, Applications, and Services.

[35] Aly H., Youssef M. New insights into wifi-based device-free localization. Proceedings of the 2013 ACM Conference on Pervasive and Ubiquitous Computing Adjunct Publication.

[36] Joshi K., Bharadia D., Kotaru M., Katti S. WiDeo: Fine-grained Device-free Motion Tracing using RF Backscatter. Proceedings of the 12th USENIX Symposium on Networked Systems Design and Implementation (NSDI 15).

[37] Scheuner J., Mazlami G., Schöni D., Stephan S., De Carli A., Bocek T., Stiller B. Probr-a generic and passive WiFi tracking system. Proceedings of the 2016 IEEE 41st Conference on Local Computer Networks (LCN).

[38] Berruet B., Baala O., Caminada A., Guillet V. (2020). An evaluation method of channel state information fingerprinting for single gateway indoor localization. J. Netw. Comput. Appl..

[39] Shen Z., Zhang T., Tagami A., Jin J. (2021). When RSSI encounters deep learning: An area localization scheme for pervasive sensing systems. J. Netw. Comput. Appl..

[40] Dang X., Cao Y., Hao Z., Liu Y. (2020). WiGId: Indoor Group Identification with CSI-Based Random Forest. Sensors.

[41] Li S., Li X., Lv Q., Tian G., Zhang D. WiFit: Ubiquitous Bodyweight Exercise Monitoring with Commodity Wi-Fi Devices. Proceedings of the 2018 Ubiquitous Intelligence &Computing.

[42] Wang H., Zhang D., Ma J., Wang Y., Wang Y., Wu D., Gu T., Xie B. Human Respiration Detection with Commodity Wifi Devices: Do User Location and Body Orientation Matter?. Proceedings of the 2016 ACM International Joint Conference on Pervasive and Ubiquitous Computing.

[43] Wu D., Zhang D., Xu C., Wang H., Li X. (2017). Device-Free WiFi Human Sensing: From Pattern-Based to Model-Based Approaches. IEEE Commun. Mag..

[44] Zhang D., Wang H., Wu D. (2017). Toward Centimeter-Scale Human Activity Sensing with Wi-Fi Signals. Computer.

[45] Zhang F., Zhang D., Xiong J., Wang H., Niu K., Jin B., Wang Y. (2018). From Fresnel Diffraction Model to Fine-grained Human Respiration Sensing with Commodity Wi-Fi Devices. Proc. ACM Interact. Mob. Wearable Ubiquitous Technol..

[46] Mouly M., Pautet M.B., Foreword By-Haug T. (1992). The GSM System for Mobile Communications.

[47] Niu K., Zhang F., Xiong J., Li X., Yi E., Zhang D. Boosting Fine-Grained Activity Sensing by Embracing Wireless Multipath Effects. Proceedings of the 14th International Conference on Emerging Networking EXperiments and Technologies.

